# Variation in Mortality and Ageing Rate in a Fast‐Paced Species: Insights From 24 Years of Hazel Dormouse (*Muscardinus avellanarius*) Data

**DOI:** 10.1002/ece3.71440

**Published:** 2025-06-11

**Authors:** Thomas Bjørneboe Berg, Fernando Colchero, Owen R. Jones, Lene Sanderhoff, Rimvydas Juškaitis

**Affiliations:** ^1^ Research Department Natural History Museum, Naturama Svendborg Denmark; ^2^ PopBio Department of Biology, University of Southern Denmark Odense Denmark; ^3^ Department of Primate Behaviour and Evolution Max Planck Institute for Evolutionary Anthropology (MPI‐EVA) Leipzig Germany; ^4^ Department of Mathematics and Computer Science University of Southern Denmark Odense Denmark; ^5^ SDU Climate Cluster University of Southern Denmark Odense Denmark; ^6^ Geo & Bio Science Centre South Faaborg Gymnasium Faaborg Denmark; ^7^ Nature Research Centre Vilnius Lithuania

**Keywords:** ageing rates, age‐specific mortality, life expectancy, survival

## Abstract

Recent research has found that, among some mammal species, differences in environmental conditions among populations of the same species drive changes in infant and juvenile mortality, but not in the rate of senescence, also known as the rate of ageing. Although this pattern has been confirmed in primates and some carnivores, it remains untested on other taxonomic groups with faster life histories, such as rodents. Here, we analysed age‐specific survival in Hazel Dormouse, using a 24‐year capture‐mark‐recapture data set from Lithuania. We used Bayesian survival trajectory analysis (BaSTA) and tested different models of age‐specific mortality. The population has experienced three distinct demographic phases—increasing (1999–2006), declining (2007–2014) and stable‐low abundance (2015–2022). We divided the dataset into these three periods to assess changes in survival over time. During all three periods, the life expectancy of males was larger than that of females, contrary to the general mammalian trend of higher female survival. Differences in survival among the three periods were primarily due to changes in age‐independent mortality and ageing rates, but not due to changes in juvenile mortality. Our findings support the notion that the low variance rate of ageing is limited to species with slow life histories. However, they also suggest that rodents, even those like the Hazel Dormouse which can reduce exposure to external threats, can substantially modulate their ageing rates in response to environmental variation.

## Graphical abstract

A Hazel dormouse population in Lithuania has undergone three distinct phases each spanning 7 years across 24 years—increasing (1999–2006), declining (2007–2014) and low abundance (2015–2022). During all three periods, male life expectancy was larger than that of females. These differences in survival were primarily due to changes in age‐independent mortality and, to a lesser degree, changes in aging rates.

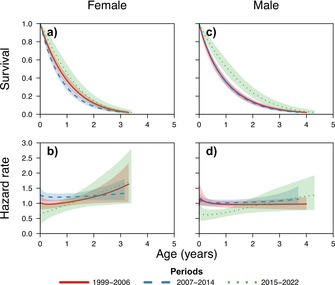

## 1 Introduction

Demographic rates, such as survival, are shaped by a multitude of mechanisms, from changes in environmental conditions to genetic and physiological differences among individuals in a population (Kendall and Fox 2002; Araya‐Ajoy et al. 2018). Importantly, these processes can affect the age trajectory of the risk of death and the resulting age‐specific survival trajectory in different ways (Kendall and Fox 2002). Among mammals, the risk of death or age‐specific mortality is commonly bathtub‐shaped, where mortality from birth to the age of sexual maturity decreases monotonically and, after maturity, increases during the adult period (Ronget et al. 2020). This monotonic increase in mortality after sexual maturity, known as actuarial senescence, results from a gradual decline in adult bodily functions (Kirkwood and Melov 2011). It has been proposed that, as environmental conditions become more adverse, the rate of senescence (the speed of change in mortality with age) should increase, thus accelerating ageing (Boonekamp et al. 2014; Tidière et al. 2016). However, recent evidence suggests that, in some long‐lived species of mammals, changes in environmental conditions affect juvenile survival and the overall level of mortality, but ageing rates change comparatively less (Colchero et al. 2021). It is unclear whether this is a generalised trend among mammals, or if it might be determined by the position of the species along the slow‐fast life history continuum (Gaillard et al. 1989, 2016). Species on the slow end, like elephants and killer whales (Ward et al. 2009), tend to reduce variability in adult survival at the expense of reproduction, while those on the fast end, like voles (*Microtus*), favour reproduction over adult survival (Gaillard et al. 1989; Oli 2004). Furthermore, some slow‐paced species are known to buffer adult survival, thus reducing its variability in response to the environment, while at the fast‐end species may preferentially buffer juvenile survival or fertility (Oli 2004; Hilde et al. 2020). Still, it is unclear whether this demographic buffering also produces higher variability in ageing rates of fast‐paced species, or if the low variance in ageing rate is universal among mammals.

A recent study on multiple populations of seven species of primates found that, as conditions became harsher, both juvenile mortality and the overall level of mortality increased, but ageing rates remained unchanged (Colchero et al. 2021). The authors described this seemingly low change in ageing rates under different environmental conditions as the ‘invariant rate of ageing hypothesis’ better reformulated as the ‘low‐variance rate of ageing hypothesis’. However, this low variability in ageing rates between populations of the same species is not universal (Cayuela et al. 2021, 2022; da Silva et al. 2022; Reinke et al. 2022). For instance, da Silva et al. (2022) found that turtles and tortoises may significantly reduce ageing rates in zoos compared to the same species living in the wild. Mazzetto et al. (2022) found higher age‐dependent gene regulation in wild than in captive individuals of a short‐lived fish, which resulted in accelerated ageing in the wild. Among rodents, senescence can vary depending on multiple factors, such as dietary restrictions, hormones and their interactions. Simons et al. (2013) showed that dietary restrictions in rats and mice can lead to delayed senescence without affecting the physical vulnerability of the individual. Even pituitary deficiencies resulting in low levels of growth hormone leading to dwarfism, have shown robust lifespan extensions among both female and male mouse models (Garratt 2020). However, it is still unclear whether these effects result in a change in the ageing rates (i.e., the speed of senescence) between populations of the same species.

Rodents provide a valuable model system to test hypotheses on the effect of the environment and population period on age‐specific mortality and ageing rates in fast‐paced species due to their relatively short generation times and their sensitivity to fluctuations in the environment (food availability, climate). This sensitivity to environmental conditions has been associated with an increase in the variation in survival and even ageing rates in rodents (Korslund and Steen 2006; Reed and Slade 2009; Turbill and Prior 2016). Given its semi‐arboreal lifestyle and its ability to hibernate that results in extended longevity compared to other small rodent species (Juškaitis 2014a), the Hazel Dormouse ( *Muscardinus avellanarius* ) provides a unique study case to explore the variation in ageing rates in a small mammal. There is some evidence that environmental conditions can affect different aspects of mortality and senescence of the Hazel Dormouse. For instance, Tidière et al. (2016) found that baseline mortality of the Hazel Dormouse decreased in zoos compared to the wild, while the onset of senescence started earlier among males living in zoos but was delayed among females. However, it is still unclear how these conditions may affect ageing rates in this long‐lived small rodent.

Hazel dormice living in the northern part of their geographic distribution face large seasonal and year‐to‐year variations in food availability during their active period (April–October) (Juškaitis et al. 2016). It is dependent on high‐quality and nutrient‐rich food resources that vary over the course of their active period (Juškaitis et al. 2016). This variation in food resources is largely associated with weather and climatic factors influencing their food supply, which in turn can affect fat reserves for hibernation, as well as juvenile and adult mortality (Pretzlaff and Dausmann 2012; Juškaitis 2014b; Turbill and Prior 2016). Notably, previous studies have shown that the average adult mortality probability was higher in females than in males, while juvenile mortality was highly dependent on time of birth within a season (Juškaitis 2014a). Importantly, the Dormouse shows sexual differences in behaviour across their active season as females bear the cost of parental care, hence having a higher energy demand than males during the breeding season, which may explain the higher mortality in females (Wells et al. 2022; Vaughan et al. 2011). Thus, contrary to most mammals whereby females have higher survival (Lemaître, Ronget, et al. 2020), the Hazel Dormouse seems to show male‐biased survival.

To examine how sex‐ and age‐specific mortality and ageing rates in the Hazel Dormouse vary under different population scenarios, we explore their temporal trends using 24 years of capture‐mark‐recapture data from Lithuania. As in other species with a fast life history (sensu Stearns 1983), we expect that sex‐ and age‐specific mortality‐related parameters will be sensitive to changes in environmental conditions.

We hypothesise that, despite their considerable longevity for a small rodent, Hazel Dormice will show high variation in ageing rates in response to environmental change as expected from fast living species, while we expect males to have higher survival than females.

## 2 Materials & Methods

### 2.1 Study Site

The 60 ha study site is part of a circa 3000 ha commercial forest tract in the Šakiai district of south‐western Lithuania (55°03′ N, 23°04′ E). The forest consists of mixed stands of approximately 70‐year‐old deciduous trees (including birch [ *Betula pendula* and *B. pubescens* ], pedunculate oak [ *Quercus robur* ], black alder [ *Alnus glutinosa* ] and ash [*Fraxinus excessior*] and coniferous trees [Norway spruce *Picea abies* ] with an understory of hazel [ *Corylus avellana* ], glossy buckthorn [ *Frangula alnus* ], bird cherry [*Padus avium*] and dwarf honeysuckle [ *Lonicera xylosteum* ]). Within the 60 ha study site, different forest management practices (small‐scale clear‐felling, thinning of regrowth, selective felling) were carried out throughout the entire study period. There were no significant changes in forest composition and intensity of forest management practices that could have influenced dormouse survival during the study period.

### 2.2 Capture‐Mark‐Recapture (CMR)

Hazel Dormice are arboreal during their active period. In the study site, 272 standard wooden nest boxes for small hole‐nesting birds were evenly distributed 3–4 m above ground in a grid system with 50 m intervals during 1999–2022. Nest box inspections were carried out twice each month during April–October. Upon capture, every individual was sexed, weighed and marked with aluminium rings with individual numbers (inner diameter—2.5 mm, height—3.0 mm). Dormice were considered adults if they had survived at least one hibernation. Unmarked young‐of‐the‐year individuals were distinguished from adults by their lower body weight, greyer fur colouration, narrower tails and earlier moulting time (Juškaitis 2006, 2014a).

The 24 years of CMR covering 1999–2022 resulted in 11,869 captures and individual marking of 4382 individuals (Table S1). These markings allowed us to identify every recaptured individual throughout its life to produce the capture‐mark‐recapture matrix required for the survival analysis. This dataset is among the most comprehensive long‐term data using CMR on small rodents.

### 2.3 Bayesian Survival Trajectory Analysis

**FIGURE 1:**
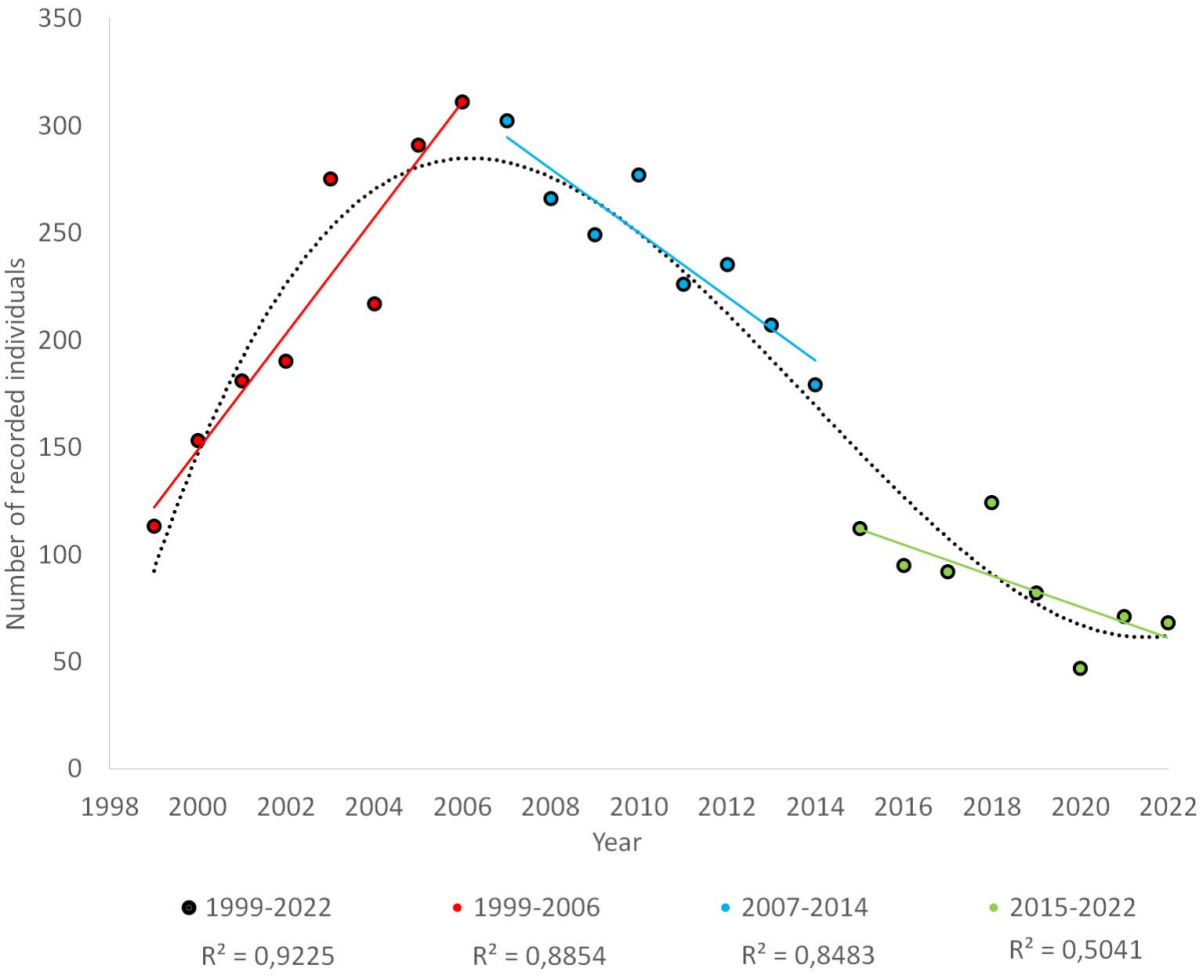
Number of recorded hazel dormouse during their active season April–October.

We divided the data into three time periods based on the shape of the broad population trends over the 24 years, where the first period consisted of a period of population increase (1999–2006), followed by a second period of population decline (2007–2014), and the third and last period of small but stable population size (2015–2022) (Figure 1 and Table S1). We carried out Bayesian survival trajectory analyses (BaSTA) for these three periods to draw inferences on age‐specific survival (Colchero and Clark 2012; Colchero et al. 2012). We used the available information on individual sex and times of birth and the few times of death as well as the capture‐recapture matrix to construct the analysis matrix required for BaSTA. Although births are easily recorded, data on death is difficult to obtain. In total, only 22 individuals out of 4367 were found dead in or next to a nest box (11 juveniles plus one adult female and seven juveniles plus three adult males). Eight of these recorded deaths could be assigned to natural causes, while the remaining 14 incidences were caused by predation by Pine Marten ( *Martes martes* ) or intraguild predation by Yellow‐necked Mouse ( *Apodemus flavicollis* ). Therefore, we included columns for the minimum and maximum times of birth and death when these were unknown based on the longevity of the species (maximum 6 years) and on the strong philopatry of the individuals in the population. As such, we were able to establish that, if an individual was not detected after 2 years of its last recapture, then it would have died within this time interval. Thus, the maximum time of death was the last recapture year plus 2 years, except when the last recapture occurred within a year of the last year of the study (i.e., 2023). Including this information helps bound the estimation of ages at death within biologically plausible ages, while it helps improve the estimation of the mortality parameters.

To make inferences on age‐specific mortality for each sex and period, we tested different mortality functions, all of which had the same initial (i.e., juvenile) mortality given by the competing risks model.

(1) $$\mu \;\left(x\right)=\exp\;\left(a_{0}-a_{1}x\right)+c+\mu_{a}\left(x\right)$$

where *a*_0_ ∈ (−∞, ∞) and *a*_1_, *c* > 0 are mortality parameters to be estimated, while function $\mu_{a}\left(x\right)$ accounts for the adult part of the mortality. Note that this general function includes an initial exponentially declining function representing infant and juvenile mortality, controlled by parameters *a*_0_ and *a*_1_ in Equation (1), while parameter *c* has commonly been described as driven by age‐independent sources of mortality.

For the adult part of the mortality, we used three different functional forms for the hazard rate or mortality function, namely Gompertz (1825) with

(2) $$\mu_{a}\;\left(x\right)=\exp\;\left(b_{0}+b_{1}x\right),$$

where *b*_0_ ∈ (−∞, ∞) and *b*_1_ > 0 are adult mortality parameters to be estimated. Note that the combination of the juvenile mortality, age‐independent mortality and Gompertz is commonly known as the Siler mortality model (Siler 1979), which has been shown to appropriately reflect the age trajectory in mortality for most mammals (Lemaître, Pavard, et al. 2020). The Gompertz function in Equation (2) assumes that mortality increases exponentially with age after sexual maturity, governed by parameters *b*_0_ and *b*_1_. Specifically, parameter *b*_1_ is a measure of senescence (ageing) rate (Ronget et al. 2020; Colchero et al. 2021).

The second adult model we tested was the Weibull model (Pinder III et al. 1978) given by the power function:

(3) $$\mu_{a}\left(x\right)=b_{0}b_{1}^{b_{0}}x^{b_{0}-1},$$

where $b_{0},b_{1}>0$. Note that this power mortality function can produce increasing but decelerating mortality with age when parameter *b*_0_ < 2. Finally, the third model we tested was the logistic mortality model (Pletcher 1999), given by

(4) $$\mu_{a}\left(x\right)=\frac{\exp\left(b_{0}+b_{1}x\right)}{1+\frac{e^{b_{0}}}{b_{1}}b_{2}\left(e^{b_{1}x}-1\right)},$$

where *b*_0_ ∈ (−∞, ∞), and *b*_1_ > 0 and $b_{2}\geq 0$. This model produces a logistic shaped mortality function and, when parameter *b*_2_ = 0, it converges to the Gompertz model.

The cumulative risk of death is given by

(5) $$U\left(x\right)=\int_{0}^{x}\mu \left(t\right)\mathrm{dt}$$

while the cumulative survival function, from which survivorship curves are produced, is calculated as

(6) $$S\left(x\right)=\exp\left[-U\left(x\right)\right]$$

The cumulative distribution function of ages at death is simply *F*(*x*) = 1 − *S*(*x*), and the probability density function (PDF) of ages at death is *f*(*x*) = $\mu \left(x\right)$ *S*(*x*), for *x* ≥ 0.

The Bayesian implementation of BaSTA uses MCMC with Metropolis‐Hastings (Metropolis et al. 1953; Hastings 1970) for sampling unknown mortality parameters and uncertain times of birth and death. We ran 10 parallel chains for 60,000 iterations, with a burn‐in of 10,001 and thinning every 50 iterations. We used the Gelman and Rubin (Gelman et al. 2013) potential scale reduction factor (Rhat) to evaluate model convergence. We selected the model with the lowest deviance information criterion (DIC, Spiegelhalter et al. 2002; Millar 2009), which combines a measure of goodness of fit and a penalisation term based on the effective number of parameters.

From the thinned mortality parameter chains of the selected model, we calculated posterior densities of summary statistics, such as life expectancy. Life expectancy at birth, which provides a precise estimate of the expected average age at death in a population, is calculated as

(7) $$e_{0}=\int_{0}^{\infty }S\left(x\right)\mathrm{dx}.$$

Given that different adult mortality models could be selected, we calculated posterior densities of ageing rates at ages *x* = 2, 4, 6, as

(8) $$\frac{d}{\mathrm{dx}}\log\mu \left(x\right)=\frac{1}{\mu \left(x\right)}\frac{d}{\mathrm{dx}}\mu \left(x\right).$$

Note that the estimates in Equation (8) are analogous to the commonly used estimation of ageing rates derived from the Gompertz *b*_1_ parameter (Ronget et al. 2020; Colchero et al. 2021).

We then used Kullback–Leibler discrepancies (KLD) (Kullback and Leibler 1951) to quantify the differences in the juvenile and age‐independent mortality parameters (first two terms in Equation (1)), as well as in life expectancy and ageing rates between the three periods for both sexes. The KLDs are calculated as

(9) $$\mathrm{KLD}=\int_{-\infty }^{\infty }p_{j}\left(x\right)\log\left[\frac{p_{j}\left(x\right)}{p_{k}\left(x\right)}\right]\mathrm{dx}$$

where *p*_*j*_(*x*) is the posterior density for a given variable, say life expectancy, in period *j* and *p*_*k*_(*x*) is the posterior density for the same variable in period *k*. This is a measure of entropy that allows us to estimate the amount of information lost if we predict the value of the variable of interest in period *k* from the posterior density of the same variable in period *j*. If both posterior densities are equal, then KLD = 0, and thus, there is no loss of information and the prediction is exact, whereas as KLD becomes larger, the amount of information loss increases and our ability to predict the variable in period *k* from that in period *j* declines steeply. As KLDs are bound in the interval [0, ∞), we used a calibration based on McCulloch (1989) that limits them to the interval [0, 1], improving interpretability. Here, a value of 0 implies that there is no loss of information (i.e., that both posterior densities are equal), and a value of 1 implies a complete loss of information (i.e., that the posterior densities have no overlap). Note that values above 0.75 can be assumed to show evidence of differences between parameter values.

## 3 Results

Based on the actual number of recorded and marked individuals across the months of April–October, the population showed large changes in size and demographic structure during the 24‐year study period (1999–2022) (Figure 1 and Table S1).

### 3.1 Survival Analyses

**FIGURE 2:**
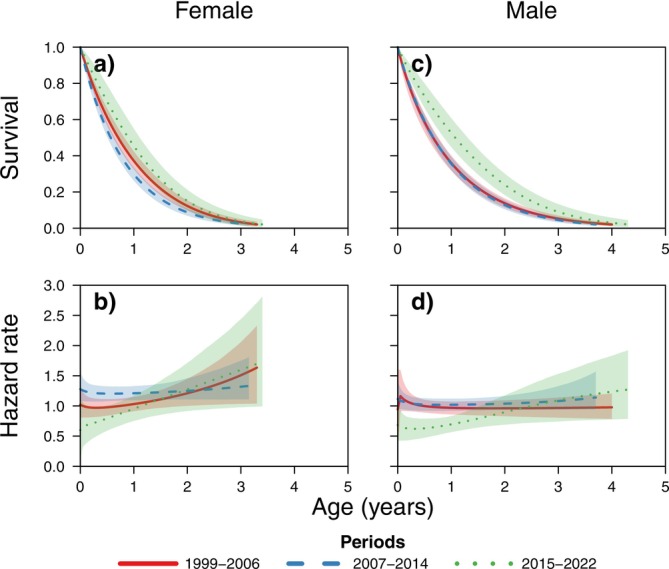
Estimated age‐specific survival and mortality for females and males in the three periods (1999–2006, 2007–2014 and 2015–2022). The lines show the mean estimate and the polygons show the 95% credible intervals.

We found important differences in age‐specific mortality within and between sexes during the three periods (Figure 2). Mortality was lowest during the last period for both sexes, and highest during the declining middle period. Contrary to our expectation, the best model was not always the typical mammalian Siler model with the adult Gompertz mortality, but it varied depending on the period and within sexes (Table 1). Females had Gompertz adult mortality during the first two periods (1999–2006, 2007–2014), and Weibull mortality during the last period. The best model for males varied between all three periods: with Weibull, then Gompertz and finally logistic. Notably, male age‐specific mortality was lowest during the stable low period (2015–2022) and highest during the decline period (2007–2014). We provide the estimated mortality parameters for females and males in Tables S2, S3.

**TABLE 1:** Deviance information criterion (DIC) for the three models tested for each sex and period.

Sex	*N*	Period	Gompertz	Weibull	Logistic
Female	504	1999–2006	**1254.36**	1297.02	1271.40
	616	2007–2014	**1305.80**	1339.27	1323.37
	175	2015–2022	495.77	**492.71**	498.55
Male	566	1999–2006	1354.34	**1350.08**	1357.31
	576	2007–2014	**1341.15**	1360.83	1348.65
	211	2015–2022	657.14	652.90	**649.74**

*Note:* The model with lowest DIC (i.e., selected) is highlighted in boldface.

Importantly, we found that males had consistently longer life expectancies than females, with 95% credible intervals only markedly overlapping in the rapid growth period (Table 2). Across the whole study period, the shortest life expectancy occurred during the decline period (females 0.82 years, males 0.96 years) while the longest life expectancy occurred during the stable low period (females 1.1 years, males 1.35 years).

**TABLE 2:** Posterior means, standard errors (SE) and lower and upper 95% credible intervals (Lower and Upper) of life expectancy and ageing rates at different ages per sex and period.

Sex	Variable	Period	Mean	SE	Lower	Upper
Female	Life	1999–2006	0.956	0.048	0.867	1.060
	Expectancy	2007–2014	0.818	0.039	0.746	0.897
		2015–2022	1.100	0.089	0.940	1.280
	Ageing rate	1999–2006	0.182	0.093	0.0003	0.356
	Age 2	2007–2014	0.039	0.055	−0.015	0.186
		2015–2022	0.241	0.115	0.035	0.494
	Ageing rate	1999–2006	0.307	0.184	0.003	0.749
	Age 4	2007–2014	0.091	0.114	−0.004	0.392
		2015–2022	0.154	0.086	0.023	0.363
	Ageing rate	1999–2006	0.414	0.301	0.007	1.230
	Age 6	2007–2014	0.208	0.310	−0.001	1.140
		2015–2022	0.113	0.066	0.018	0.277
Male	Life	1999–2006	0.992	0.049	0.897	1.090
	Expectancy	2007–2014	0.963	0.045	0.877	1.050
		2015–2022	1.350	0.092	1.170	1.540
	Ageing rate	1999–2006	0.001	0.034	−0.058	0.079
	Age 2	2007–2014	0.027	0.040	−0.015	0.132
		2015–2022	0.230	0.117	0.021	0.479
	Ageing rate	1999–2006	0.009	0.029	−0.026	0.091
	Age 4	2007–2014	0.105	0.145	−0.003	0.510
		2015–2022	0.091	0.080	0.003	0.294
	Ageing rate	1999–2006	0.011	0.029	−0.016	0.095
	Age 6	2007–2014	0.284	0.415	−0.0002	1.510
		2015–2022	0.035	0.061	−0.002	0.211

**FIGURE 3:**
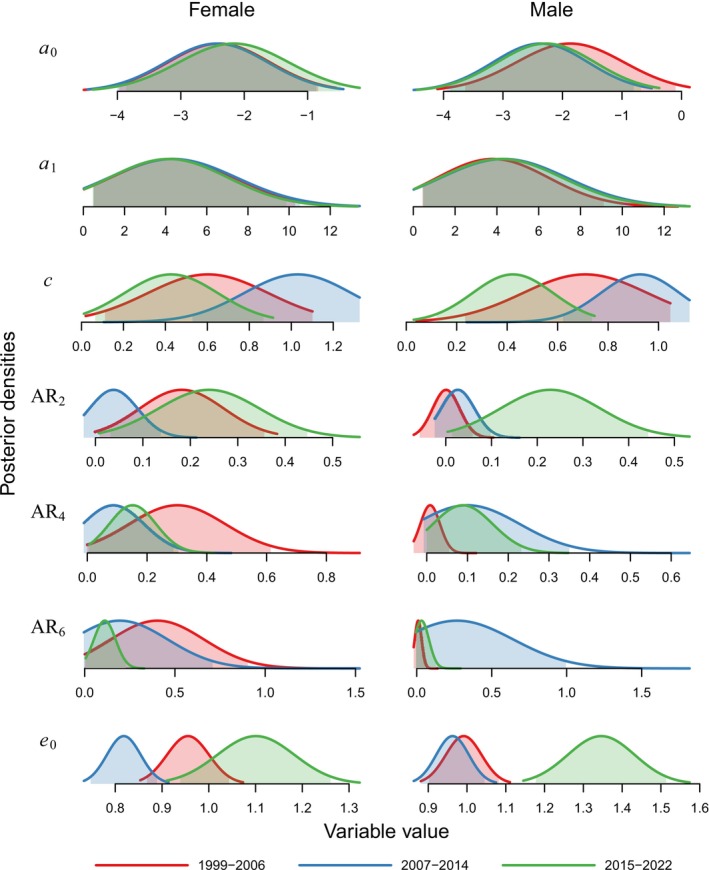
Posterior densities of juvenile and age‐independent parameters (*a*_0_, *a*_1_ and *c*), of ageing rates (AR) at 2, 4 and 6 years of age, and life expectancy (*e*_0_) for each sex and period.

We found that most of the differences in mortality trajectories between the three periods were driven by large changes in the mortality parameter *c* and by differences in ageing rates at different periods, as confirmed by the high calibrated Kullback–Leibler discrepancies (KLDs) (Table 3 and Figure 3). Similarly, life expectancies varied considerably between periods for both sexes, with a notable exception in males between the first and second periods. Interestingly, the KLDs also show that juvenile mortality for both sexes (determined by the *a*_0_ and *a*_1_ parameters) remained remarkably stable among the three periods (KLD < 0.2).

**TABLE 3:** Calibrated Kullback–Leibler discrepancies of juvenile and age‐independent mortality parameters (a0, a1 and c), life expectancy and ageing rates (AR) at different ages per sex between pairs of periods.

sex	Periods	*a*_0_	*a*_1_	c	Life expect	AR age 2	AR age 4	AR age 6
Female	Per 1—Per 2	0.00	0.00	**0.77**	**1.00**	**0.82**	0.67	0.48
	Per 1—Per 3	0.04	0.01	0.30	**0.90**	0.26	0.65	**0.90**
	Per 2—Per 3	0.05	0.01	**0.91**	**1.00**	**0.95**	0.50	0.69
Male	Per 1—Per 2	0.16	0.03	0.47	0.17	0.38	0.70	**0.87**
	Per 1—Per 3	0.11	0.01	**0.80**	**1.00**	**0.98**	**0.79**	0.34
	Per 2—Per 3	0.01	0.00	**0.95**	**1.00**	**0.95**	0.24	**0.80**

*Note:* Values above 0.75 are highlighted in boldface. The periods are as follows: Per 1 = 1999–2006, Per 2 = 2007–2014, Per 3 = 2015–2022.

## 4 Discussion

We set out to examine differences in mortality trajectories among different periods of population development (growth, decline and stability) and between the sexes. Our BaSTA analyses showed that differences in the mortality trajectories were largely determined by differences in the so‐called age‐independent mortality parameter (*c*) and in ageing rates (Figure 3). Note that the models we tested here are described as competing risk models; this is that they are constructed under the assumption that different types of risks act additively on the overall mortality (Patricio and Missov 2024). The *c* parameter represents a constant risk with age, and thus, it is associated with mortality factors that are expected to affect all ages equally, which could include climatic or human‐driven changes in the composition of their habitat (e.g., forest management). These factors may increase exposure to predators or reduce available resources, thus increasing overall starvation, all of which affect all individuals irrespective of their age.

The differences in ageing rates show that, contrary to slow species, such as primates (Colchero et al. 2021), and despite their relatively long lifespan for a small rodent, ageing rates in the Hazel Dormouse can still substantially change as a function of changes in population density and environmental conditions. Importantly, due to its semi‐arboreal lifestyle and habit of hibernation, the Hazel Dormouse is less exposed to common sources of mortality than most other rodents of the same size, such as those with more ground‐dwelling lifestyles and year‐round activity like in the genera *Apodemus* and *Clethrionomys*, which both have shorter lifespans than the Hazel Dormouse (Juškaitis 2014a; Nowak 1999; Kryštufek 2017). Although this reduced mortality may explain the Hazel Dormouse's relative longevity, ageing rates still vary as expected among fast‐lived species. However, it is still unclear whether these adaptations to hibernation and arboreal life translate into relatively lower variation in ageing rates in Hazel dormice compared to other small rodents. The impact of such protective adaptations on the evolution of longevity is well documented across many types of species (Finch 1990; Healy et al. 2014), which act by tipping the balance of resource allocation towards somatic maintenance and away from reproduction in populations where age‐independent mortality is lower. The results can be dramatic: for instance, hibernating species, like members of the Gliridae family, can live twice as long as their non‐hibernating counterparts (Juškaitis 2014a; Weigl 2005).

Despite the potentially protective life period during hibernation, Juškaitis (2014b) found that seasonal mortality within the Hazel Dormouse in Lithuania is generally lower during summer (27.1%–51.9%) than during winter (42.5%–80%). The Hazel Dormouse is affected by climate in different ways across the two main seasons, winter dormancy and summer activity. Warmer temperatures during summers may have less effect on mortality than during the winter hibernation, due to increased energy consumption during summer compared with a constant cold and dry winter scenario. We believe that the seemingly important role of weather conditions, as also stated by Combe et al. (2023), may explain why most of the differences in mortality are through the age‐independent parameter and ageing rates, and are not reflected in the juvenile parts of the mortality function. In a recent study covering five populations of Hazel Dormouse, four in the UK as well as the present population in Lithuania across the years 2006–2015, Combe et al. (2023) found population density, precipitation and increased winter temperature to be the variables that negatively affect population growth rates and survival. As food is highly affected by weather variables, this indirect climatic effect on the Hazel Dormouse is an additive effect to focus on in future studies.

The monitored population of Hazel Dormouse went through a substantial decline of 72% from the peak observed number of adults in 2004 to the all‐time low in 2020. A similar dramatic decline of 78% was reported from the UK over the period from 1994 to 2020 (Scopes et al. 2023). It is, though, not necessarily a result of a permanent population decline, because an ongoing long‐term study shows that Hazel Dormouse populations may remain in low but comparatively stable abundance for many years (Unpublished data Juškaitis R and Mortensen RM). We found that life expectancy ranges from 0.82 to 1.1 years for females and 0.96 to 1.35 years for males over the course of the study period (Table 2). Dormouse populations do fluctuate over consecutive years. In the present study, annual changes in the total number of recorded individuals were up to 50%. This is far lower than fluctuations known from other rodent species like field vole and lemmings that can reach fluctuations of more than a factor of 10 in density (Lambin et al. 2000; Ehrich et al. 2001).

Adult Hazel Dormice are regarded to be highly stationary, leaving the dispersal to the young of the year. In a study on different populations in Italy, Bani et al. (2017) showed that dispersal is also likely to be sex‐biased, as females tend to disperse more than males in a fragmented landscape. As adult Hazel Dormice show a philopatric behaviour, Juškaitis (2014b) regarded that failure to recapture previously marked adult individuals could be an incidence of mortality. Juškaitis (2014b) found that during 13 years of surveys (2000–2012), the proportion of individuals not recaptured was significantly higher in females than in males, indicating a sex‐biased summer mortality resulting from depletion of fat reserves after hibernation as well as predation. Time of birth also affects the mortality during hibernation due to lack of sufficient fat reserves, as revealed by Juškaitis (2014a) that late‐born juveniles, which did not reach a body weight of approximately 15 g before entering hibernation, showed a significantly higher mortality than those that gained more than 15 g before hibernation. For those hazel dormice that do reach the necessary body fat threshold before hibernation, the main factors affecting winter survival are climate and predation (Combe et al. 2023; Juškaitis 2014a). Given that the Hazel Dormouse hibernates for up to 7 months, its ability to benefit from mast years is much lower compared to other rodent species like the yellow‐necked mouse and bank vole that do not hibernate. The yellow‐necked mouse and, to some degree, the bank vole can maintain their food consumption during winter through food storage and thus, in contrast to the Hazel Dormouse, positively affect their winter survival and extend their breeding season by giving birth to more litters (Wolff 1996; Mappes 1998; Butet and Delettre 2011). During a mast year, one yellow‐necked mouse was found to give birth to litters in both January and February (Berg unpublished data).

As expected, survival was lowest for both sexes during the decline period (Figure 2a,c). More strikingly, and contrary to the common trend in mammalian longevity research where females typically outlive males (Ronget et al. 2020), our findings indicate that males had consistently longer life expectancies than females (Table 2). Notably, the general mammalian trend of females outliving males has been explained as the result of sex differences in the intensity of sexual selection (Bateman 1948; Promislow 1992). Males are expected to allocate resources heavily on competition for mates and territorial defence, whereas females allocate to offspring production and care (Bonduriansky et al. 2008; Brooks and Garratt 2017; Marais et al. 2018). The result is that, in general, the costs to survival of these strategies are expected to be higher for males, particularly for polygynous species (Clutton‐Brock and Isvaran 2007; Tidière et al. 2015). In comparison with males, female Hazel Dormouse need more time for foraging (even during the day) during pregnancy and lactation periods to meet increased energy demands. This increased activity elevates their risk of predation. In autumn, late‐breeding females face a narrower window to accumulate fat reserves before hibernation, and food conditions decline as the season progresses (less suitable food remains). Consequently, these females often accumulate insufficient fat reserves which may lead to higher winter mortality. It is likely that, for the dormouse, the intensity of sexual selection is relatively low among males, whereas the cost of reproduction for females associated with lactation, nursing and offspring protection is disproportionally high, particularly during years with harsh environmental conditions (Wells et al. 2022).

Interestingly, adult male mortality during the last period followed a typical logistic form (Figure 2d). Logistic mortality curves can result from a selection effect on individual heterogeneity, whereby frailer individuals (those with a higher hazard rate) die earlier, producing the apparent asymptote in the mortality curve (Vaupel and Missov 2014). A similar process may be at play in females, where adult mortality followed a decelerating Weibull function. The deceleration in mortality with age can also be in response to individual heterogeneity, albeit the effect is less pronounced than in males. It remains unclear whether these decelerating and even asymptotic mortality patterns are the result of individual heterogeneity and why these patterns only emerge during the last period when the population density is at its lowest. Notably, this last period also exhibited the lowest overall mortality and highest life expectancies for both sexes. This may suggest that, at low densities, the effect of individual heterogeneity becomes more pronounced in both sexes, possibly due to reduced competition or, in males, a decline in agonistic interactions.

## 5 Conclusions

In contrast to the general trend in mammals that females have a longer life expectancy than males (Ronget et al. 2020), we show the opposite situation is the case for the Lithuanian population of Hazel Dormouse. During all three study periods (increase, decline and steady low period), the life expectancy of males was greater than that of females (between 7.6% and 26.9% longer, depending on the period). The differences in mortality trajectories and resultant life expectancies among the three periods were primarily due to changes in age‐independent mortality and ageing rates. Notably, these results are consistent with predictions from the demographic buffering hypothesis, which states that adult survival, and by extension, its components, such as ageing rates, is less buffered against environmental variation (Oli 2004; Hilde et al. 2020). However, variation in ageing rates among fast‐lived species remains poorly understood. Further research on the mortality of such species is crucial if we are to understand the proximate and evolutionary mechanisms that control the rate of ageing.

## Author Contributions

**Thomas Bjørneboe Berg:** data curation (equal), project administration (lead), writing – original draft (equal), writing – review and editing (equal). **Fernando Colchero:** conceptualization (lead), data curation (lead), formal analysis (lead), validation (equal), writing – original draft (equal), writing – review and editing (equal). **Owen R. Jones:** formal analysis (supporting), validation (equal), writing – original draft (equal), writing – review and editing (equal). **Lene Sanderhoff:** writing – original draft (supporting). **Rimvydas Juškaitis:** data curation (equal), investigation (lead), methodology (lead), resources (lead), writing – review and editing (supporting).

## Conflicts of Interest

The authors declare no conflicts of interest.

## Supporting information


Tables S1–S3.

## Data Availability

The data and code to reproduce the results in this paper are openly available in Zenode at https://doi.org/10.5281/zenodo.15227880.
