# Supplementary material for: Variation in Mortality and Ageing Rate in a Fast‐Paced Species: Insights From 24 Years of Hazel Dormouse (Muscardinus avellanarius) Data
Source: Ecol Evol. 2025 Jun 11;15(6):e71440. doi: 10.1002/ece3.71440 (PMC12152644; doi:10.1002/ece3.71440)
Supplement: Supplementary file 1 — Tables S1–S3. [file ECE3-15-e71440-s001.zip › ece371440-sup-0001-TableS3.docx]

Table S3 Estimated male mortality parameters for each period. The columns show the selected adult mortality model, the parameters for the corresponding model as well as the recapture probability, π, the Mean estimate, the standard error (SE), the lower and upper 95% credible intervals (Lower, Upper), and the potential scale reduction value for model convergence (Rhat).

| **Sex** | **Period** | **Model** | **Parameter** | **Mean** | **SE** | **Lower** | **Upper** | **Rhat** |
| --- | --- | --- | --- | --- | --- | --- | --- | --- |
| Male | 1999-2006 | **WE** | *a*_0_ | -1.87 | 0.96 | -3.78 | -0.14 | 1.00 |
|  |  |  | *a*_1_ | 3.88 | 2.88 | 0.16 | 10.90 | 1.00 |
|  |  |  | *c* | 0.71 | 0.26 | 0.08 | 1.02 | 1.01 |
|  |  |  | *b*_0_ | 1.42 | 0.72 | 0.60 | 3.26 | 1.00 |
|  |  |  | *b*_1_ | 0.26 | 0.24 | 0.01 | 0.88 | 1.00 |
|  |  |  | π | 0.92 | 0.02 | 0.87 | 0.96 | 1.00 |
|  | 2007-2014 | **GO** | *a*_0_ | -2.37 | 0.86 | -4.12 | -0.79 | 1.00 |
|  |  |  | *a*_1_ | 4.39 | 3.18 | 0.09 | 11.50 | 1.00 |
|  |  |  | *c* | 0.92 | 0.18 | 0.35 | 1.10 | 1.03 |
|  |  |  | *b*_0_ | -5.68 | 3.34 | -13.30 | -0.63 | 1.00 |
|  |  |  | *b*_1_ | 0.62 | 0.47 | 0.03 | 1.77 | 1.00 |
|  |  |  | π | 0.91 | 0.03 | 0.85 | 0.95 | 1.00 |
|  | 2015-2022 | **LO** | *a*_0_ | -2.28 | 0.88 | -4.09 | -0.64 | 1.00 |
|  |  |  | *a*_1_ | 4.25 | 3.13 | 0.14 | 11.60 | 1.00 |
|  |  |  | *c* | 0.42 | 0.17 | 0.06 | 0.71 | 1.00 |
|  |  |  | *b*_0_ | -2.48 | 0.82 | -4.22 | -1.08 | 1.00 |
|  |  |  | *b*_1_ | 1.07 | 0.43 | 0.30 | 1.99 | 1.00 |
|  |  |  | π | 1.15 | 0.60 | 0.15 | 2.44 | 1.00 |
|  |  |  | *a*_0_ | 0.84 | 0.04 | 0.75 | 0.91 | 1.00 |
