# Supplementary material for: Variation in Mortality and Ageing Rate in a Fast‐Paced Species: Insights From 24 Years of Hazel Dormouse (Muscardinus avellanarius) Data
Source: Ecol Evol. 2025 Jun 11;15(6):e71440. doi: 10.1002/ece3.71440 (PMC12152644; doi:10.1002/ece3.71440)
Supplement: Supplementary file 1 — Tables S1–S3. [file ECE3-15-e71440-s001.zip › ece371440-sup-0001-TableS1.docx]

Table S1. Recorded number of 4382 individuals over the years 1999-2022 divided into sex and age groups.

| **Year** | **Juveniles** | **Adult males** | **Adult females** | **Total** |
| --- | --- | --- | --- | --- |
| **1999** | 85 | 24 | 13 | 122 |
| **2000** | 119 | 28 | 16 | 163 |
| **2001** | 115 | 38 | 31 | 184 |
| **2002** | 140 | 32 | 27 | 199 |
| **2003** | 211 | 35 | 37 | 283 |
| **2004** | 126 | 46 | 50 | 222 |
| **2005** | 222 | 40 | 43 | 295 |
| **2006** | 228 | 52 | 28 | 308 |
| **Sum** | 1246 | 295 | 245 | 1776 |
| **2007** | 218 | 38 | 41 | 297 |
| **2008** | 172 | 42 | 43 | 257 |
| **2009** | 178 | 36 | 34 | 248 |
| **2010** | 182 | 47 | 46 | 275 |
| **2011** | 163 | 38 | 24 | 225 |
| **2012** | 169 | 32 | 36 | 237 |
| **2013** | 161 | 28 | 17 | 206 |
| **2014** | 113 | 40 | 26 | 179 |
| **Sum** | 1356 | 301 | 267 | 1924 |
| **2015** | 69 | 27 | 16 | 112 |
| **2016** | 73 | 13 | 12 | 98 |
| **2017** | 58 | 21 | 11 | 90 |
| **2018** | 78 | 25 | 20 | 123 |
| **2019** | 35 | 29 | 13 | 77 |
| **2020** | 20 | 16 | 10 | 46 |
| **2021** | 40 | 19 | 10 | 69 |
| **2022** | 31 | 25 | 11 | 67 |
| **Sum** | 404 | 175 | 103 | 682 |
| **Total** | 3006 | 771 | 615 | 4382 |
